# More Than Bar Codes: Integrating Global Standards-Based Bar Code Technology Into National Health Information Systems in Ethiopia and Pakistan to Increase End-to-End Supply Chain Visibility

**DOI:** 10.9745/GHSP-D-16-00350

**Published:** 2017-12-28

**Authors:** Liuichi Hara, Ramy Guirguis, Keith Hummel, Monica Villanueva

**Affiliations:** a Independent consultant. Formerly with the United Nations Population Fund (UNFPA), Copenhagen, Denmark.; b United States Agency for International Development, Washington, DC, USA.; c United States Agency for International Development/Ethiopia, Addis Ababa, Ethiopia.; d United States Agency for International Development/Pakistan, Islamabad, Pakistan.

## Abstract

The United Nations Population Fund (UNFPA) and the United States Agency for International Development (USAID) DELIVER PROJECT work together to strengthen public health commodity supply chains by standardizing bar coding under a single set of global standards. From 2015, UNFPA and USAID collaborated to pilot test how tracking and tracing of bar coded health products could be operationalized in the public health supply chains of Ethiopia and Pakistan and inform the ecosystem needed to begin full implementation. Pakistan had been using proprietary bar codes for inventory management of contraceptive supplies but transitioned to global standards-based bar codes during the pilot. The transition allowed Pakistan to leverage the original bar codes that were preprinted by global manufacturers as opposed to printing new bar codes at the central warehouse. However, barriers at lower service delivery levels prevented full realization of end-to-end data visibility. Key barriers at the district level were the lack of a digital inventory management system and absence of bar codes at the primary-level packaging level, such as single blister packs. The team in Ethiopia developed an open-sourced smartphone application that allowed the team to scan bar codes using the mobile phone's camera and to push the captured data to the country's data mart. Real-time tracking and tracing occurred from the central warehouse to the Addis Ababa distribution hub and to 2 health centers. These pilots demonstrated that standardized product identification and bar codes can significantly improve accuracy over manual stock counts while significantly streamlining the stock-taking process, resulting in efficiencies. The pilots also showed that bar coding technology by itself is not sufficient to ensure data visibility. Rather, by using global standards for identification and data capture of pharmaceuticals and medical devices, and integrating the data captured into national and global tracking systems, countries are able to lay the foundation for interoperability and ensure a harmonized language between global health stakeholders.

## Abstract (precis)

Bar codes can help track and trace health products in the supply chain. But to do so efficiently, they should be based on global standards rather than a proprietary system, and the captured data should be integrated into national health information systems to achieve end-to-end data visibility.

## INTRODUCTION

Low- and middle-income countries often rely on inaccurate and labor-intensive processes to manage key health commodity supply chains.^1^ However, recent innovations in supply chain technology have helped improve the efficiency of commodity acquisition, management, and delivery systems, thus reducing stock-outs and ensuring health commodities, such as pharmaceuticals and medical devices, reach the end user.^1^^,^^2^ The challenge has been finding a consistent, effective, and inclusive approach to increasing supply chain data visibility, as the availability of quality and timely data often varies greatly within developing countries.

Supply chain visibility is “the awareness of, and control over, specific information related to product orders and physical shipments, including transport and logistics activities, and the statuses of events and milestones that occur prior to and in-transit.”^3^ Data visibility requires a robust data collection system that is agile and incorporates and synchronizes the needs of various partners into a single multitiered responsive system that begins with the production of the health product (drug or device) and ends with it in the hands of the end user.^3^

Adopting global standards and using bar code technology can help countries to address accuracy, interoperability, and timeliness of data across supply chain levels; achieve end-to-end (E2E) data visibility; and directly help improve forecast and quantification as well as improve procurement and supply coordination among the donor agencies.

To that end, the United States Agency for International Development (USAID) DELIVER PROJECT and the United Nations Population Fund (UNFPA) worked with the governments of Ethiopia and Pakistan to design and test pilot studies to validate the conclusion that automatic identification and data capture (AIDC) systems could be used to improve E2E supply chain visibility of health commodities.^1^^,^^2^ AIDC is a method of identifying items, collecting data, and transmitting that data directly electronically—in these pilots, through bar codes.

## ACHIEVING END-TO-END SUPPLY CHAIN DATA VISIBILITY

AIDC is a key tool for improving product visibility in the global supply chain. While there are various approaches used to achieve AIDC, bar codes and radio frequency identification are the most commonly used.

Automatic identification and data capture systems are a key tool for improving product visibility in the global supply chain.

Leveraging AIDC provides an organization the ability to track and trace tangible assets in real-time or near real-time. The International Organization for Standardization defines track and trace as a “means of identifying every individual material goods or lots or batch in order to know where it has been (track) and where it is (trace) in the supply chain”.^4^ Unique product identification linked with the item's batch number or serial number and expiration date are rapidly becoming a prerequisite to track and trace health care products to create an E2E supply chain.^5^

With an efficient track and trace system, an organization or a country can effectively address complex integrity issues, such as distribution of counterfeit pharmaceutical products and theft or diversions of shipments. This can only be achieved by improving the E2E supply chain data visibility. Using a bar coding system that complies with global standards is crucial to maintain an organization's supply chain integrity and to safeguard public health.

## USING GLOBAL STANDARDS

As part of their formative research, UNFPA and the USAID DELIVER PROJECT identified a clear need to raise awareness of existing global standards, such as bar codes, and the value of integrating their use into the health care sector. Global standards for identification, capture, and sharing are provided by GS1, a “neutral, not-for-profit, international standards organization that develops global standards to improve the efficiency and visibility of supply chains across industries.”^3^

UNFPA and the USAID DELIVER PROJECT identified a clear need to raise awareness of existing global standards, such as bar codes, and the value of integrating their use into the health care sector.

Although bar codes have been used to improve inventory tracking in low- and middle-income countries, there is limited documentation of cases that have led to the adoption of bar code systems beyond the pilot phase or to realize their value across all the systems in the supply chain.^6^ This may be explained by a lack of adoption of internationally accepted standards for AIDC among the key stakeholders—such as donors, pharmaceutical companies, logistics providers, regulatory agencies, and implementing partners—and resulted in each donor or provider developing a proprietary solution specific to a funded project.

However, there is growing acceptance among many donors, countries, and the private sector regarding the value of adopting a global standard for product identification and bar codes to improve supply chain efficiency. This is because GS1 global standards are product-agnostic and provide a framework to scale onto all products across the different health programs—such as childhood vaccines and HIV/AIDS—and build the foundation for interoperability. In effect, the use of global standards help to improve patient safety and reduce exposure to supply chain integrity issues.

## APPLYING THEORY TO PRACTICE: THE JOURNEY

Pakistan and Ethiopia conducted proof of concepts for an E2E supply chain data visibility approach using bar codes with logistics information dashboards. The 2 cases are discussed individually in this section and their findings and lessons learned are compared in the following reflections section.

### Pakistan

**FIGURE 1.**
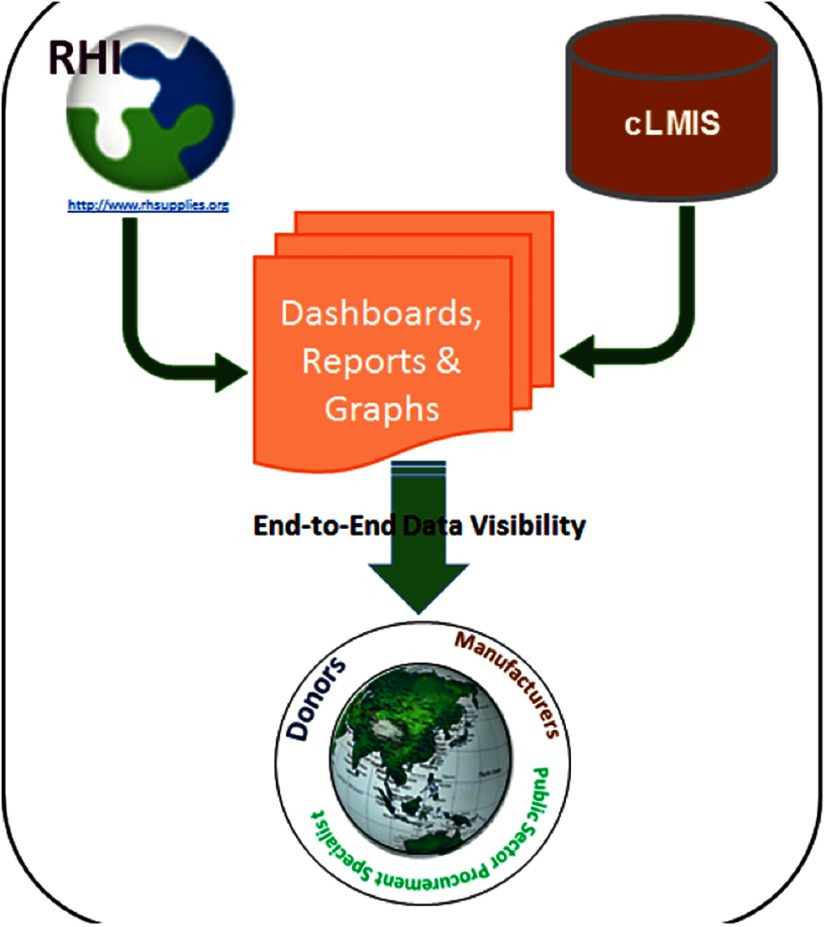
Pakistan End-to-End Dashboard Structure

Abbreviations: cLMIS, contraceptive logistics management information system; RHI, Reproductive Health Interchange.

The USAID DELIVER PROJECT in Pakistan developed a web interface with global procurement information through the Reproductive Health Interchange (https://www.unfpaprocurement.org/rhi-home), and combined the interface with Pakistan's contraceptive logistics management information system, which tracks the distribution and stock status of family planning commodities across the entire country (Figure 1). This system informs federal and provincial procurement actions.

Pakistan was not new to the idea of using bar codes, as it already implemented use of proprietary bar codes for inventory management of contraceptive supplies in 2012.^1^ However, the pilot conducted in 2015 emphasized the value of transitioning from proprietary tracking methods to bar codes based on global standards.

The transition allowed the Pakistan team to leverage the original 2-dimensional DataMatrix bar codes preprinted by global manufacturers as opposed to printing new bar codes at the central warehouse. In order to read the original bar codes, the team invested in a new Windows mobile-based Motorola MC9200 handheld optical scanner, as the previous handheld scanner was limited to 1-dimensional linear bar codes only.

The transition from proprietary tracking methods to global standards-based bar codes allowed Pakistan to leverage the original bar codes that were preprinted by global manufacturers, as opposed to printing new bar codes at the central warehouse.

**FIGURE 2.**
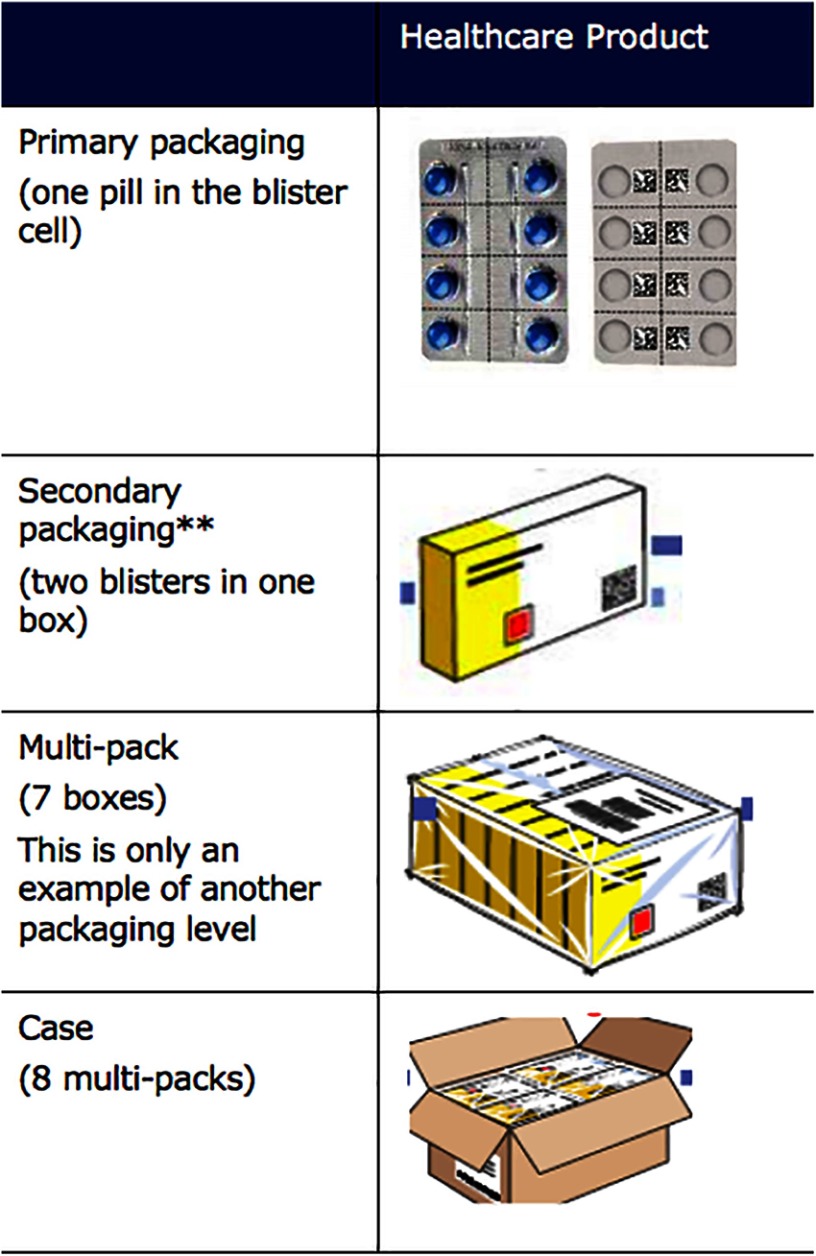
GS1 Package Hierarchy Examples

The Pakistan bar code pilot experience highlighted 2 key aspects for future work in E2E supply chain data visibility: (1) the lack of an inventory management information system at the district level posed a challenge to consolidating the captured bar code data; and (2) products arriving into the districts were primary-level packaging—for example, single blister packs—that lacked bar codes (Figure 2). Therefore, extending information system installation and applying bar codes at the primary package will be required if tracking and tracing is to be extended down to the district level.

### Ethiopia

Similarly, the USAID DELIVER PROJECT in Ethiopia followed the approach of developing a web interface with the project's “My Commodities” system and the Reproductive Health Interchange to merge the global procurement information with the national warehouse management software (called the Health Commodity Management Information System). My Commodities provides registered users with shipment information of health supplies, contraceptives, condoms, personal protective equipment for avian influenza control, antimalarials, and other commodities.

**FIGURE 3.**
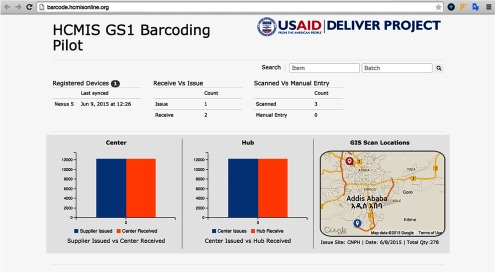
Ethiopia End-to-End Dashboard Sample

The Ethiopia pilot test was an important milestone, as it expanded beyond the Pakistan experience. The Ethiopia team developed an open-sourced smartphone application using the built-in CMOS image sensors (the camera technology) commonly found on standard Android smartphones. Bar code scanning was performed through the CMOS camera via the mobile application, which then pushed the captured data from the bar code to Ethiopia's data mart and E2E dashboard. Real-time tracking and tracing was demonstrated from the central warehouse to 2 major distribution points: the Addis Ababa distribution hub and 2 subsequent health centers. Furthermore, the Android smartphone's GPS coordinates were integrated with a geographical information system to display transactional information—the issuance and receipt of products—onto a Google Map (Figure 3).

**Figure:**
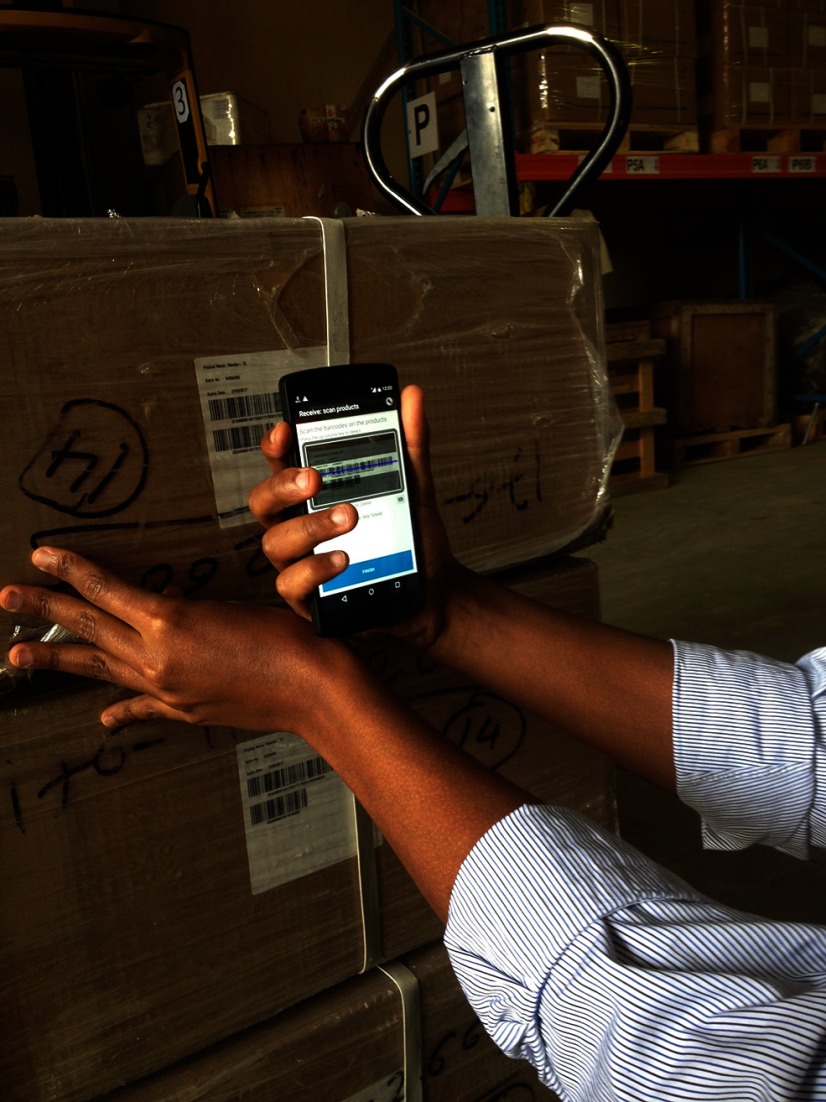
In Ethiopia, a team member at the Addis Ababa central warehouse uses a mobile phone app to scan bar codes on shipping boxes when receiving incoming goods. © 2015 L Hara

Lastly, UNFPA sent the bar code requirements to the supplier in advance of on-the-ground testing. This enabled the Ethiopia team to enter the standardized unique product information—the global trade item number, batch number, and expiry date—into the national health information system prior to receipt of incoming goods. The time for scanning and recording the digital information was measured and is summarized in Table 1. This preparation allowed the team to validate and cross reference the cargo received at the central warehouse in real-time once they scanned the bar codes. This pilot demonstrated the immediate benefits that could be achieved by using globally standardized bar codes and integrating data systems; namely, by reducing manual steps for recording inbound and outbound goods and reducing the chance of human error via misentry of data.

**TABLE 1.** Time for Scanning and Recording the Digital Bar Code Information, Ethiopia Pilot Test

Location	Transaction	No. of Scans	Time to Scan and Record Information From Bar Code	Comments
Central store	Inbound receipt	13 tertiary-level shipper boxes	3 minutes and 37 seconds	Recorded time included physically moving the packages and obstacles
Central store	Inbound receipt	63 secondary-level packages	24 minutes and 38 seconds	Software optimization made during the test; used smartphone torch feature and improved scanning technique based on experience at the central store
Addis Ababa distribution hub	Inbound receipt	13 tertiary-level shipper boxes	2 minutes	
Addis Ababa distribution hub	Inbound receipt	50 secondary-level packages	15 minutes	Quantity split between 2 local health centers
Addis Ababa distribution hub	Outbound to local health center	230 secondary-level packages	Less than 25 minutes

The Ethiopia pilot demonstrated the immediate benefits that could be achieved by using globally standardized bar codes and integrating data systems; namely, by reducing manual steps for recording inbound and outbound goods and reducing the chance of human error via misentry of data.

## REFLECTIONS

While the 2 country experiences were distinct, they both showcased the potential and challenges in realizing E2E visibility. A comparison of the 2 pilots is summarized in Table 2.

**TABLE 2.** Comparison of the Pakistan and Ethiopia Pilot Tests

Features	Pakistan	Ethiopia
In-country electronic logistics management information system	cLMIS	HCMIS
Bar coding	Transition from proprietary to GS1 bar codes	GS1 bar codes, from the outset
E2E dashboard	Achieved by integrating RHI with cLMIS and incorporating data from bar code scanning	Achieved by integrating RHI with HCMIS and incorporating data from bar code scanning
Serialization	Not part of the pilot test	Serialization was done at the secondary package level
Scanning approach	Handheld optical scanners	Open-sourced Android smartphone app developed locally (HCMIS barcode scanner)
Where track and trace was pilot tested	Central Warehouse and Supplies, Karachi to Lahore district store	Central warehouse to Addis Ababa distribution hub to Woreda health center and Nefas Silk Lafto health center
Result	Full E2E track and trace was not achieved due to lack of inventory management system at the district level and lack of bar codes at the primary unit level	Full E2E track and trace via digital scanning demonstrated to the exact number of packages distributed between the 2 health centers

Abbreviations: cLMIS, contraceptive logistics management information system; E2E, end-to-end (supply chain); HCMIS, Health Commodity Management Information System; RHI, Reproductive Health Interchange.

The Ethiopia experience more realistically demonstrated what full E2E bar code track and trace could look like: A digital cargo manifest identifier is encoded in the bar codes at the tertiary-level shipper boxes. Upon arrival of the shipment, the receiving party can digitally authenticate the cargo manifest by simply scanning the bar codes and automatically recording the data into the dashboard. Encoded data about the products from the bar codes on the secondary-level packages can continue to be scanned as the products move downstream inside the country so that track and trace can be achieved down to the last mile of the supply chain.

The Pakistan experience, on the other hand, clearly demonstrated the barriers at the lower levels of supply chain, such as the availability of a consistent digital information system and use of bar codes at the lowest product unit level. Depending on the product origin and product presentation, such as blister packs or vials, bar coding at the lowest unit level may be easier for some products than others. It is important to communicate this issue to the original manufacturer to begin dialogue for bar coding at the primary unit level.

However, without a proper digital information system to receive the scanned data, the value of AIDC greatly diminishes. Therefore, an ecosystem is needed that combines bar codes and the appropriate digital information system(s) and processes in place to ensure that scanned and recorded information are used for proper decision making.

Without a proper digital information system to receive the scanned data, the value of AIDC greatly diminishes.

Another point that needs consideration is deciding which scanning approach is most appropriate for the country context. In Ethiopia, the team used a locally developed open-sourced smartphone application, while in Pakistan the team procured new handheld optical scanners. Although both options served their intended purpose, we recommend that countries consider their national technology capacity, and then choose the approach and type of investment—short- or long-term—that best suits a country's needs. A brief comparison between smartphone and handheld scanners has been compiled in Table 3.

**TABLE 3.** Comparison Between Smartphone and Handheld Scanners

Approach	Pros	Cons
Smartphone scanner	Flexibility to customize and update app software Ability to leverage existing personal smartphones Ability to adopt or adapt the app (open-source)	Poor ergonomic design for scanning Slower scan speed rate Function depends on mobile penetration in the country Several mobile apps, which can be confusing for the user Higher battery burn rate to smartphone
Handheld scanner	Faster scan speed rate Good ergonomic design for scanning	Stable funding is needed to procure, maintain, and/or upgrade handheld scanners at all distribution touch points
Hybrid handheld scanner connected to a smartphone via Bluetooth (as an alternative for future consideration)	Lower cost than traditional handheld scanner Faster scan speed rate Good ergonomic design for scanning Can leverage smartphone app software	Higher battery burn rate to smartphone

While the penetration of mobile services is on the rise in developing countries, according to the International Telecommunication Union, mobile cellular subscriptions for 2015 was reported at 43 per 100 people in Ethiopia and 67 per 100 people in Pakistan.^7^ Based on the 2015 Pew Research Center analysis, Ethiopia and Pakistan were rated as having 2 of the lowest smartphone ownership rates globally (4% and 11%, respectively).^8^ Ethiopia has the added challenge of being captive to only 1 operator, which supports 42.1 million mobile connections in the country.^9^ In contrast, Pakistan has 8 operators supporting 127.9 million mobile connections in the country.^10^ These are important factors to consider when incorporating mobile technology into track and trace designs. If a country has insufficient mobile network coverage, then handheld optical scanners, which do not rely on mobile networks, should be considered. Along the same logic, if a high number of operators support a vibrant mobile network coverage, smartphones may be the best option to perform the scanning.

## CONCLUSION

The collective experience from the Pakistan and Ethiopia pilots highlights the importance of adopting bar codes as part of a global standardized system for product identification and data capture that serves as a foundation for interoperability and data sharing that is essential to achieve end-to-end data visibility in the supply chains.

The pilots demonstrated the value of implementing an automated logistic management information system based on global standards and using bar code technology to improve the efficiency of the supply chain operation, address the data quality issues, and achieve near real-time data visibility. This ultimately helps to ensure that patients have continuous and consistent access to high-quality medicines at the right time and right place.

The pilots demonstrated the value of implementing an automated logistic management information system based on global standards and using bar code technology to improve the efficiency of the supply chain operation, address the data quality issues, and achieve near real-time data visibility.

While there is still considerable work to do before countries can reach optimal E2E data visibility, the results from these and related pilots indicate that we can reach this goal by adopting the same global standards and practice for public health supply chains.

## Notes

**Peer Reviewed**

**Funding:** UNFPA, USAID.
